# Supplementary material for: Value-added scores show limited stability over time in primary school
Source: PLoS One. 2022 Dec 28;17(12):e0279255. doi: 10.1371/journal.pone.0279255 (PMC9797061; doi:10.1371/journal.pone.0279255)
Supplement: S1 Table — (DOCX) [file pone.0279255.s004.docx]

Supplemental Material

**S4 Table. Covariance Table of VA Scores with Different Outcome Domains (Mathematics and Language) over Time (2017 to 2019).**

|  | 2017 Mathematics | 2017 Language | 2019 Mathematics | 2019 Language |
| --- | --- | --- | --- | --- |
| 2017 Mathematics | 850.074 | 501.911 | 289.991 | 275.873 |
| 2017 Language | 501.911 | 850.074 | 185.754 | 317.185 |
| 2019 Mathematics | 289.991 | 185.754 | 850.074 | 397.259 |
| 2019 Language | 275.873 | 317.185 | 397.259 | 850.074 |

*Note*. Covariances are calculated on the basis of *n*= 7,016 elementary school students’ VA scores. The VA scores of 2017 and 2019 were informed by data from 2015 and 2017, respectively.
